# Assessment of Visual Acuity, Refraction Changes, and Proptosis in Different Ages of Patients with Thyroid Diseases

**DOI:** 10.1155/2012/643275

**Published:** 2012-11-06

**Authors:** J. Jankauskiene, D. Jarusaitiene

**Affiliations:** Eye Clinic, Medical Academy, Lithuanian University of Health Sciences, Mickeviciaus 9, 44307 Kaunas, Lithuania

## Abstract

*Objective*. The aim of the study was to assess visual acuity, refractive status, and eye proptosis in children and young adults with Graves' disease. *Material and Methods*. We have done investigations of visual acuity, refraction, and eye proptosis in 16 children, 14 teenagers, and 49 adults with Graves' disease at Eye Clinic of Lithuanian University of Health Sciences Medical Academy. Data were compared with 14 children, 14 teenagers, and 35 adults of similar age without the same diseases (control group). *Results*. In the present study we observed a significant decrease of visual acuity in teenagers (0.73 ± 0.18, *P* = 0.001) and adults (0.7 ± 0.16, *P* = 0.001) with Graves' disease. Myopia was ascertained more frequent in teenagers (42.8 percent) and adults (44.9 percent) with Graves' disease. In our study exophthalmometer values were higher in children (15.94 ± 1.98 mm, *P* = 0.003), teenagers (17.28 ± 2.99 mm, *P* = 0.01), and adults (18.05 ± 2.91 mm, *P* = 0.001) in comparison with the controls. *Conclusions*. The data we have found suggest that Graves' disease in children, teenagers, and adults has influence on vision acuity, refraction, and eye proptosis.

## 1. Introduction

Patients with thyroid disease (thyrotoxicosis, diffuse, or nodular goiter) can manifest different eye lesions: eye proptosis, changes in vision, and refraction characterized by a multifactor mechanism of development. Endocrine ophthalmopathy (Graves' ophthalmopathy) presents as an autoimmune process which affects soft ocular tissue, eye muscles, cornea, and optic nerve [1]. These symptoms and signs typically peak at approximately 6 to 24 months following the onset of Graves' disease and have a significant impact on visual function [2]. Inflammatory mediators released by lymphocytes in the orbit drive orbital fibroblasts to produce glycosaminoglycans. This causes interstitial edema and enlargement of extraocular muscles within the tight confines of the orbit, with secondary mechanical effects [3]. The clinical manifestations of Graves' ophthalmopathy can be understood from a mechanistic point of view. Proliferation of retrobulbar cells (fatty and connective tissue cells lacrimal gland cells) and their infiltration with lymphocytes, plasmic cells, macrophages, mucopolysaccharides and the excretion of various inflammatory factors as well as an increased extent of extraocular muscles have an influence on the development of exophthalmos [4].

Endocrine ophthalmopathy of children and teenagers is rare, and its clinical signs are less well defined in comparison with the adult population [5]. In children and teenagers with thyrotoxicosis eyelid manifestations are frequent, and eyelid retraction is most common [6]. Endocrine ophthalmopathy incidence in Europe has been reported to be 0.8 per 100,000 children per year, the female-to-male ratio being about 3 : 1 up to 5 : 1 [7].

The current literature on refractive changes in endocrine ophthalmopathy is limited. Chandrasekaran et al. (2006) reported the myopic shift following orbital decompression in progressive endocrine ophthalmopathy [8]. It has been established that receptors of thyroid hormones are present in all tissues and organs of the body except the brain, spleen, and testicles so it is quite credible that tonicity of ciliary muscles (smooth fibers) also depends on the activity of thyroid gland. That is why hyperthyroidism can cause refractive changes, mostly those of myopia [9]. Considerable increase of thyroid hormones may have influence on the short sightedness [10].

There are few articles about refraction, visual acuity, and eye proptosis in children, teenagers, and adults with thyroid diseases, and data are contradictory. That is why we wanted to analyze the influence of Graves' disease and on visual acuity, refraction, and proptosis in children, teenagers, and adults.

The aim of the study is to assess visual acuity, refractive status, and eye proptosis in children, teenagers, and adults with Graves' disease.

## 2. Material and Methods

We have done investigations of visual acuity, refraction, and eye proptosis in 16 children from 6 to 14 years (average 8.25 ± 3.9 year), 14 teenagers from 14 to 18 years (average 17.1 ± 3.1 year), and 49 adults from 18 to 63 years (average 43.2 ± 3.8 year) with Graves' disease at Eye Clinic of Lithuanian Health Sciences University Medical Academy. 75 patients (94.9%) were nonsmokers, and four patients (5.1%) were smokers. The diagnosis of Graves' disease was based on typical clinical findings and thyroid function tests (thyrotropin (TSH), free thyroxine (FT4) and free triiodothyronine (FT3) levels, thyroid peroxidase antibodies (TPOAb)).

Inclusion criteria are patients with Graves' disease. Exclusion criteria: patients with ocular or other endocrine and systemic diseases.

The data were compared with the data of control group obtained from 14 healthy children, 14 teenagers, and 35 adults of similar age.

The ocular examination included measurements of visual acuity (VA) and slit-lamp investigation. Exophthalmometry was performed by single observer (one measurement) with Hertel's exophthalmometer. Objective refraction was investigated using retinoscopy. Myopia was characterized as refractive error as −0.50 spherical equivalent or more, hyperopia as +0.50 spherical equivalent or more.

Statistical analysis was conducted using statistical SPSS software package (Version 16.0). The following statistical characteristics were expressed as a mean value and standard deviation (SD). The statistical difference between patients and control groups was tested with the Student's *t*-test. For groups with abnormal distribution Mann-Whitney *U* test for independent samples was used. A *P* value less than 0.05 was considered statistically significant.

## 3. Results

Visual assessment showed that in 18.7 percent of children with Graves' disease achieved visual acuity (VA) equal 1.0. It has been ascertained that in children with Graves' disease the mean of visual acuity was 0.8 ± 0.14, control −0.89 ± 0.11. There was no significant difference between the children control and patients groups (Table 1). Visual acuity in teenagers was 0.73 ± 0.18, control −0.92 ± 0.08 (*P* = 0.001). It has been ascertained that, in adults with Graves' disease, visual acuity was 0.7 ± 0.16 and was worse than in the control group (0.9 ± 0.1), (*P* = 0.001).

4 (25 percent) of children with Graves' diseases aged 6–12 years were emmetropic, 10 (71.42 percent) were hyperopic, and 2 (12.50 percent) were myopic. The mean spherical equivalent in children with Graves' diseases was +1.17 ± 1.13, rangeing from −0.5 to +2.0 D, (Table 2). There was no significant difference between the mean spherical equivalent in children control and patients groups. The mean spherical equivalent in children control group was +0.64 ± 0.94 D, 4 children of control group (28.6%) were emmetropic, 2 of them (14.3%) were myopic, and 8 (57.1%) were hyperopic. 4 (28.6%) teenagers with Graves' disease had no refractive changes, 4 (28.6%), hyperopic, and 6 (42.9%), myopic. The mean spherical equivalent was −1.05 ± 2.31 D, ranging from −4.25 to +1.75 D. 7 (50%) teenagers of the control group had no refractive changes, and 7 (50%), hyperopic. The mean spherical equivalent in teenagers control group was +0.32 ± 0.34 D, (*P* = 0.04). In adult patients 11 (22.45%) were without refractive errors, 16 (32.6%), hyperopia, and myopia was in 22 (44.9 percent). The mean spherical equivalent in adult patients with Graves' disease ranged from −5.75 to +2.5 D and was −1.83 ± 2.25 D comparing with the mean spherical equivalent in adult control group (+0.31 ± 0.77 D), (*P* = 0.001).

Hertel exophthalmometry in children with Graves' diseases showed that proptosis ranged from 13.0 to 19.0 mm, mean −15.94 ± 1.98 mm, and was bigger in comparison with control group (ranging from 11.0 to 17.0 mm, mean −13.43 ± 2.01 mm), (*P* = 0.003), (Table 3).

In teenagers with Graves' disease proptosis ranged from 13 to 22.5 mm, mean of proptosis was 17.28 ± 2.99 mm and it was significantly higher than in control group (ranging from 12.0 to 18.0 mm, mean −14.21 ± 1.75 mm), (*P* = 0.01).

Hertel exophthalmometric values in adults had a mean (18.05 ± 2.91 mm, ranged from 13.5 to 23.0 mm) and was significantly higher than in control group (ranging from 13.0 to 17.5 mm, mean −15.27 ± 1.23 mm), (*P* = 0.001).

## 4. Discussion

We have found few investigations on the influence of thyroid pathology on the development of myopia and visual acuity impairment, so we compared our results with those of similar studies.

Phillips et al. and Frank-Raue et al. found that the excess of thyroid hormones brought about by the mutation of receptors of thyroid hormones had an influence on the development of myopia [10, 11].

Our data showed that in children the difference of visual acuity between the control group and children with Graves' disease did not reach significance. In the present study we observed a significant decrease of visual acuity in teenagers and adults with Graves' disease.

Refractive changes in Graves' ophthalmopathy might have been affected by the immune complexes accumulated on the lateral walls of the orbit and by enlarged volume of the motor muscles of eyeball. Then, under pressure, the eyeball undergoes remodeling with the longitudinal ocular axis growing longer. Weakening of visual acuity is induced by optic nerve neuropathy brought about by the thickening of rectal eye muscles and the pressure exerted by the optic nerve at the orbital apex.

According to the findings of our study, it has been noted that teenagers and adults with Graves' disease demonstrated refractive changes. Myopia was more frequent in teenagers and adults with autoimmune thyroid pathology.

Although there is little reference to refractive change associated with Graves' ophthalmopathy reported in the literature, there are few papers that report other important refractive changes. One of them describes patients with Graves' ophthalmopathy and corneal astigmatism, which, overall, is not influenced by orbital, strabismus, or eyelid surgery. The astigmatism may possibly be caused by soft-tissue fibrosis in the superolateral orbital region [12]. Chandrasekaran et al. noted that myopic shift after decompression is consistent with mechanisms involving the posterior pole. Combination of enlarged extraocular muscles, anterior displacement of the globe, and orbital hypertension related to elevated muscle and fat volumes in endocrine ophthalmopathy flattens the posterior pole, which may produce choroidal folds [8].

Murdoch and Merriman reported that choroidal folds may be as a mechanism of acquired hypermetropia. The literature reports an acquired hypermetropia with large choroidal folds [13].

Other investigators indicated that myopia degree directly depended on the optical axis length. It has been established experimentally that when optical axis lengthens by 1 mm, myopia increases by 3D [14].

Huismans ascertained myopia in Graves' ophthalmopathy and suggests edema and infiltration of lymphocytes and plasmacytes of ciliary-body, and damage of it, pathologic-anatomical substratum of the most changes of orbitae in this disease as a possible mechanism for myopia [15]. In cases of thyroid pathology visual acuity and refractive changes develop due to the proliferation of retrobulbar cells, causing exophthalmos. One of myopia development reasons is eye-ball remodeling, along with thyroid hormones affecting the tonicity of ciliary muscle [16]. In comparing our investigation results and literature data, the same tendencies have been observed.

Autoimmune hyperthyroidism is the most common cause of juvenile thyrotoxicosis in children and teenagers. Eha et al. investigated patients under the age of 18 years (range 3–16 years, mean age 14.5 years) with Graves' disease and signs of ophthalmopathy [17]. In our study exophthalmometer values were higher in children, teenagers, and adults with Graves' disease in comparison with the controls. But in children with thyroid autoimmune disease eye proptosis was less prominent than in teenagers and adults with thyroid diseases. Earlier studies showed that exophthalmometer readings in children have changed with growth. Nucci et al. reported exophthalmometer measurements in children ranging from 3 to 10 years old and noted that exophthalmometry data during adolescence, as measurements, tend to increase with age and especially in puberty [18]. Liu et al. showed that normal exophthalmometer values in children (from 3 to 16 years old) have ranged from 14.5 mm to 16 mm. Data confirmed the prominent proptosis in childhood thyroid eye disease. Proptosis measurements ranged from 14 mm to 33 mm [19]. Therefore we agree with the observation of Nucci et al. and Liu et al., who obtained nearly similar results as ours.

## 5. Conclusion

Visual acuity in teenagers and adults with Graves' disease appeared to be worse than in persons of the control group. Myopic refractive error was ascertained more frequent in teenagers and adults with Graves' disease. Exophthalmometry values were significantly higher than in controls in children teenagers and adults with Graves' disease. The data we have found suggest that Graves' disease in children, teenagers, and adults have influence on proptosis, visual acuity, and refractive changes.

## Figures and Tables

**Table 1:** Visual acuity in children, teenagers, and adults with Graves' disease.

Persons	Patients VA	Control group VA	*P*
Children	0.8 ± 0.14	0.89 ± 0.11	0.06
Teenagers	0.73 ± 0.18	0.92 ± 0.08	0.001
Adults	0.7 ± 0.16	0.9 ± 0.1	0.001

**Table 2:** The mean spherical equivalent in patients with Graves' disease.

Persons	Patients	Control group	*P*
	The mean spherical equivalent D	The mean spherical equivalent D	
Children	+1.17 ± 1.13	+0.64 ± 0.94	0.17
Teenagers	−1.61 ± 2.39	+0.32 ± 0.34	0.01
Adults	−1.34 ± 2.37	+0.31 ± 0.77	0.001

**Table 3:** Proptosis in children, teenagers, and adults with Graves' disease.

Patients proptosis mm	Control group Proptosis mm		*P*
Children	15.94 ± 1.98	13.43 ± 2.01	0.003
Teenagers	17.28 ± 2.99	14.21 ± 1.75	0.01
Adults	18.05 ± 2.91	15.27 ± 1.23	0.001
